# Significantly elevated calcium levels and gallstone disease in a patient with familial hypocalciuric hypercalcaemia

**DOI:** 10.1530/EDM-26-0022

**Published:** 2026-09-08

**Authors:** Adam Sabo, Andrew McGovern

**Affiliations:** ^1^ North Devon District Hospital, Barnstaple, UK; ^2^ University of Exeter Institute of Biomedical and Clinical Science, Exeter, UK

**Keywords:** calcium, rare diseases/syndromes

## Abstract

**Summary:**

Familial hypocalciuric hypercalcaemia (FHH) is a rare, benign genetic disorder of calcium homeostasis caused by inactivating variants in CaSR, GNA11, or AP2S1 genes. It is typically characterised by mild, asymptomatic hypercalcaemia and low urinary calcium excretion. Differentiation from primary hyperparathyroidism (PHPT) is essential, as misdiagnosis can lead to unnecessary surgery. We report a case of FHH type 1 (FHH1) in a man in his thirties presenting with a serum calcium level of 2.97 mmol/L, which is at the severe end of the FHH spectrum. Genetic testing revealed a heterozygous CaSR nonsense variant (NM_000388.4:c.1942C>T, p.Arg648Ter) consistent with FHH1. The patient had clinically stable hypercalcaemia, which was not treated. During follow-up, he developed gallstones treated with a laparoscopic cholecystectomy. A review of the literature demonstrated that calcium levels approaching 3 mmol/L, although uncommon, have been reported in other cases of FHH1. We review the literature regarding a potential association between FHH and gallstones. This case highlights that FHH can present with relatively marked hypercalcaemia, reinforcing the need for genetic evaluation before surgery, and suggests a possible but under-explored relationship between FHH and gallstone disease.

**Learning points:**

- Clinicians should be alert to the possibility of FHH even in significant hypercalcaemia and a calcium-to-creatinine clearance ratio (CCCR) above 0.01.
- Genetic testing might increasingly become a tool for phenotype prediction in FHH.
- Whilst FHH is generally considered a benign condition, in phenotypes with a higher calcium, it may be associated with an increased risk of gallstones.

## Background

Familial hypocalciuric hypercalcaemia (FHH) is a rare genetic disorder leading to dysregulation of calcium homeostasis via increased reabsorption of calcium in the loop of Henle. Three types are recognised – FHH1, FHH2, and FHH3 – associated with variations in CaSR, GNA11, and AP2S1 genes, respectively, leading to combined prevalence of between 1 in 10,000 and 1 in 100,000. Despite varying genetic aetiology, all of these variations lead to reduced sensitivity of calcium-sensing cells in parathyroid glands and thus impairment of the negative feedback loop between calcium and parathyroid hormone (PTH) release. Inappropriate release of PTH then results in continued reabsorption of calcium in the kidneys despite existing hypercalcaemia. Additionally, reduced activity of the calcium-sensing receptors in the kidneys will increase the threshold for calcium excretion, resulting in increased tubular reabsorption and consequent hypocalciuria despite hypercalcaemia. The different types of FHH result in similar clinical presentations – mild hypercalcaemia with few to no clinical symptoms.

FHH must be differentiated from primary hyperparathyroidism (PHPT), which is a much more common cause of hypercalcaemia and can have a similar presentation, i.e. incidental identification and being asymptomatic. PHPT can present with a wide range of calcium values from very mild hypercalcaemia to calcium values in excess of 4.00 mmol/L. Hypercalcaemia in FHH is usually mild, and therefore, FHH presenting with more pronounced hypercalcaemia has a high risk of being misdiagnosed as PHPT (1).

To help differentiate between the two, calcium:creatinine clearance ratio (CCCR) and serum PTH levels are commonly used. Unlike PHPT, the PTH levels in FHH are not normally significantly raised as the physiological homeostasis settles around a new raised set point of serum calcium level resulting in normalisation of PTH. However, PTH levels in the normal range frequently occur in PHPT. Coexistent vitamin D deficiency may increase PTH levels in FHH through superimposed secondary hyperparathyroidism, potentially obscuring the distinction from primary hyperparathyroidism, and should be corrected before PTH is interpreted (2). A CCCR of above 0.01 is commonly used to exclude FHH (3).

Although delay in FHH diagnosis is unlikely to cause any significant harm to a patient, following the PHPT pathway as a result of missed diagnosis certainly can. A patient with a missed diagnosis of FHH could undergo unnecessary exploratory parathyroidectomy – a procedure with risks and no benefit in FHH.

Here, we present a case of FHH with an unusually high serum calcium at diagnosis. The case raises several clinically important questions, which we have answered through a review of the relevant literature.

## Case presentation

A man in his thirties was referred by his general practitioner to the outpatient endocrinology clinic in North Devon due to persistently elevated calcium and paraesthesia in his fingers and toes, and he was seen in the endocrinology clinic in November 2022. He was otherwise well with no medical history. He worked as a cleaner and smoked 20 cigarettes a day. Previously, he was a binge drinker but now has moderate intake of alcohol. On presentation, he used cocaine once a week. His mother also had elevated calcium, although it was uncertain whether the cause of this had been investigated.

## Investigation

**Figure 1:**
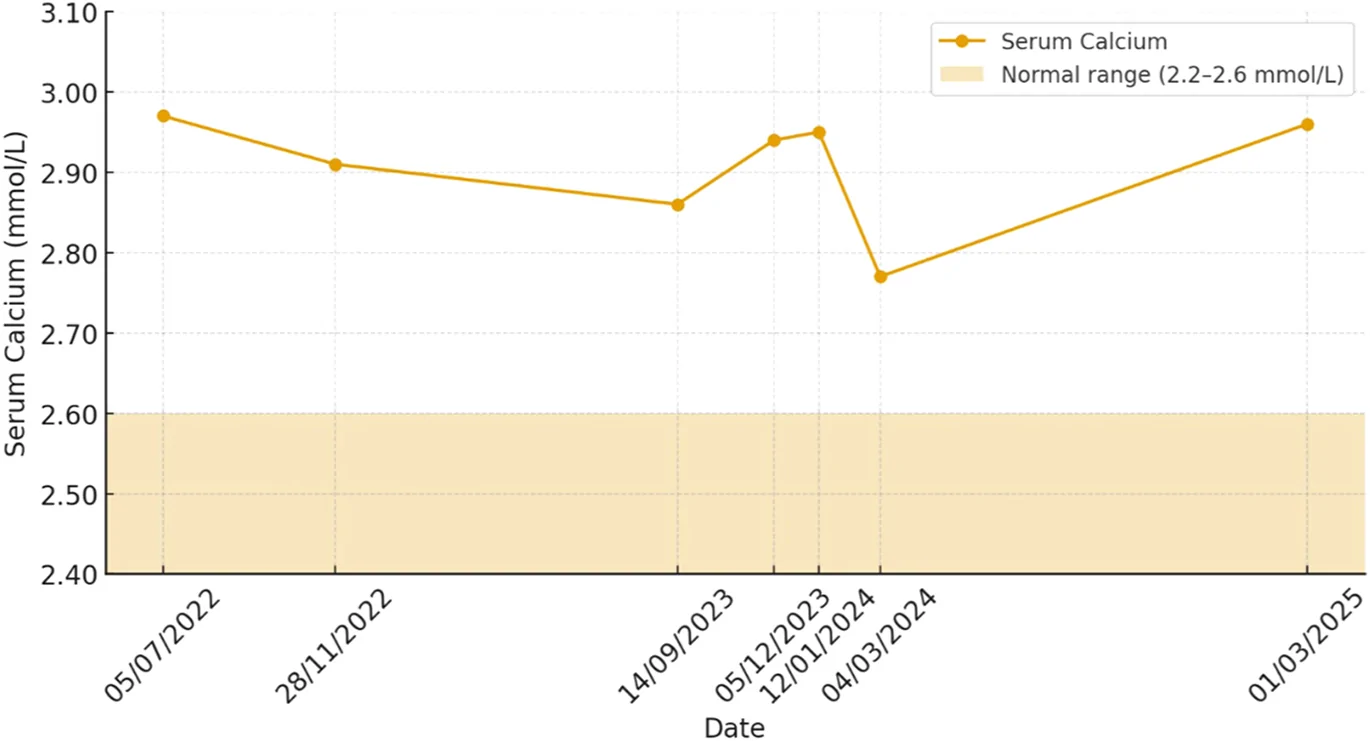
Trends in the patients’ calcium corrected for albumin at presentation and during follow-up.

On presentation to GP in July 2022, he was found to have calcium corrected for albumin of 2.97 mmol/L (Fig. 1). His TSH was normal at 1.43 mIU/L (0.27–4.20 mIU/L), and he had normal renal function, PTH 4.6 pmol/L (1.6–6.9 pmol/L), and magnesium 0.89 mmol/L (0.70–1.00 mmol/L). He was normotensive of 116/81 mmHg and had a mildly elevated BMI of 26 kg/m^2^. A 24-h urine collection was arranged and collected 3.08 L with 24 h urinary creatinine being 13.2 mmol/24 h and 24 h urinary calcium being 2.4 mmol/24 h (2.5–7.5 mmol/24 h). The resulting CCCR was 0.0053. He then underwent genetic testing and was found to be heterozygous for CASR nonsense variant NM_000388.4:c.1942C>T, p.(Arg648Ter) and diagnosed with FHH type 1.

## Treatment

We note reports of successful cinacalcet use in FHH to lower hypercalcaemia and thus manage significant symptoms, such as polydipsia, palpitations, or muscle weakness (4, 5). Current evidence for its use seems to only be supported by case reports and further investigation with randomised control trials is warranted. Our patient did not require treatment beyond dietary advice and vitamin D supplements as his symptoms were relatively mild.

## Outcome and follow-up

He went on to have calculous cholecystitis with subsequent laparoscopic cholecystectomy in 2024. He never suffered from kidney stones. On follow-up in February 2025, he had low vitamin D at 41 nmol/L (25–50 nmol/L) and was given vitamin D supplements 800 units daily. Most recent calcium in January 2025 was 2.96 mmol/L, and he continued to suffer from a mild tingling feeling, which was most pronounced in his feet and testes.

## Discussion

Our case raised several clinically important questions, which we have attempted to answer through a target review of the literature:

- FHH typically causes mild hypercalcaemia and is frequently not considered as a differential diagnosis for more pronounced hypercalcaemia. How common is it to have calcium levels of around 3 mmol/L in FHH as seen in our patient?
- Are specific variations in FHH1 associated with the degree of hypercalcaemia?
- Calcium is involved in gallstone formation. Is gallstone disease precipitated by increased serum calcium and is there known association with FHH or was the presence of gallstones in our patient unrelated to the FHH?

### How common is it to have calcium levels of around 3 mmol/L in FHH as seen in our patient?

Levels of calcium of around 3 mmol/L have mainly been reported in association with FHH3, but similar levels have sporadically been seen in FHH1 as well with our literature review giving the proportion of hypercalcaemia above 2.95 mmol/L at 7.93% in FHH1 out of 164 cases (6, 7, 8, 9). Conversely, some studies we reviewed in our literature search did not report any cases with serum calcium as high as 2.96 mmol/L despite relatively sizeable samples (10). Therefore, whilst our case represents the more extreme part of the spectrum of hypercalcaemia in FHH1, it is certainly not unique.

Several authors have noted that using a CCCR cut-off of >0.01 to exclude FHH can lead to many missed cases of FHH (7, 9). A higher threshold of 0.02 has been suggested to ensure that a higher proportion of potential FHH cases are correctly diagnosed (11).

These data suggest that clinicians should be alert to the possibility of FHH in people with calcium values around 3 mmol/L and that using a CCCR cut-off of >0.01 to exclude FHH is not completely robust. FHH should still be considered before exploratory surgery in those with a CCCR <0.02 and negative parathyroid imaging even when the calcium levels are as high as 3 mmol/L.

### Are specific variations in FHH1 associated with the degree of hypercalcaemia?

Specific nucleotide variants are indeed associated with the degree of hypercalcaemia in FHH. In particular, it appears that whether the resulting peptide is or is not degraded by the nonsense-mediated mRNA decay (NMD) pathway is significantly associated with severity of the resulting phenotype and thus calcium levels. Current evidence suggests that variations that undergo NMD result in a more severe FHH phenotype (10). Additionally, there is evidence that receptors truncated following a nonsense variation seem to produce more moderate phenotypes when compared to substitutions (8, 12).

The variation reported in our case report – NM_000388.4:c.1942C>T, p.(Arg648Ter) – is a nonsense variation predicted to escape NMD (https://nmdprediction.shinyapps.io/nmdescpredictor/). Clinical manifestation of this variation has been described before with serum calcium levels also significantly elevated (13, 14), with highest level reported being 2.89 mmol/L.

The variant identified in our patient has resulted in a more severe phenotype than would be predicted from what is known about the correlations between variants and the phenotype but is consistent with other cases with the same variant.

### Is gallstone disease precipitated by increased serum calcium and is there known association with FHH or was the presence of gallstones in our patient unrelated to the FHH?

We found no studies exploring the link between FHH and biliary pathology, such as biliary stones and cholecystitis, apart from two older clinical reviews with no statistical analysis (15, 16). Although most gallstones are cholesterol based, around 15% are calcium based (17). There are reports, mainly in transgenic mice or *in vitro*, showing that CaSR malfunction inhibits cholecystokinin (CCK) release (18, 19). Gallstone formation has been linked to both impaired gallbladder emptying and dysfunctional CCK release (20, 21). The link between the formation of gallstones and PHPT seems to be well established on the other hand, with a systematic review reporting an odds ratio of occurrence for gallstone disease of 1.77 (95% confidence interval: 1.60–1.97) in people with PHPT compared with controls (22). Taken together, these findings suggest a possible association between gallstone disease and FHH, particularly in patients with significant hypercalcaemia such as our patient. However, the patient did have some other background risk factors for developing gallstones, such as increased alcohol intake, mildly raised BMI, and smoking history, and therefore, we cannot determine a causal link between his hypercalcaemia and gallstones. Observational studies are required to confirm and further explore this potential association.

## Conclusion

High calcium values around 3 mmol/L are rare in FHH1 but can occur. Clinicians should not discount the possibility of FHH at this level of hypercalcaemia and should also be aware of increasing potential of genetics to predict FHH phenotype. We additionally propose a hypothesis that hypercalcaemia in FHH may predispose to gallstones, but further research is needed to explore this association.

## Declaration of interest

The authors declare that there is no conflict of interest that could be perceived as prejudicing the impartiality of the research reported.

## Funding

This research was supported by the National Institute for Health and Care Research (NIHR) Exeter Biomedical Research Centre (BRC). The views expressed are those of the author(s) and not necessarily those of the NIHR or the Department of Health and Social Care.

## Patient consent

Written informed consent for publication of their clinical details and/or clinical images was obtained from the patient.

## Author contribution statement

All authors made individual contributions to authorship. AM was involved in management of the patient. AS and AM were both involved in writing and submission of the manuscript.
